# Individuals of a group-living shorebird show smaller home range overlap when food availability is low

**DOI:** 10.1186/s40462-023-00427-9

**Published:** 2023-10-27

**Authors:** He-Bo Peng, Chi-Yeung Choi, Zhijun Ma, Allert I. Bijleveld, David S. Melville, Theunis Piersma

**Affiliations:** 1 Groningen Institute for Evolutionary Life Sciences (GELIFES), University of Groningen, P.O. Box 11103, 9700 CC Groningen, The Netherlands; 2 Department of Coastal Systems, NIOZ Royal Netherlands Institute for Sea Research, PO Box 59, 1790 AB Den Burg, Texel, The Netherlands; 3 Division of Natural and Applied Sciences, Duke Kunshan University, Kunshan, 215316 Jiangsu China; 4 Environmental Research Center, Duke Kunshan University, Kunshan, 215316 Jiangsu China; 5 School of Environmental Science and Engineering, Southern University of Science and Technology, Shenzhen, China; 6 Ministry of Education Key Laboratory for Biodiversity, Science and Ecological Engineering, National Observations and Research Station for Wetland Ecosystems of the Yangtze Estuary, and Institute of Eco-Chongming (SIEC), School of Life Sciences, Fudan University, Shanghai, 200433 China; 7 Global Flyway Network, c/o 1261 Dovedale Road, RD2 Wakefield, Nelson, 7096 New Zealand; 8 CEAAF Center for East Asian-Australasian Flyway Studies, Beijing Forestry University, Qinghua East Road 35, Haidian District, Beijing, 100083 China; 9 BirdEyes, Centre for Global Ecological Change at Faculties of Science and Engineering and Campus Fryslân, University of Groningen, Zaailand 110, 8911 BN Leeuwarden, The Netherlands

**Keywords:** Aggregation, Overlap, Migratory shorebirds, Waders, Food shortage

## Abstract

**Background:**

Group living animals, such as shorebirds foraging on intertidal mudflats, may use social information about where to find hidden food items. However, flocking also increases intraspecific competition for resources, which may be exacerbated by food scarcity. Therefore, although aggregation may bring benefits, it may also increase the intensity of intraspecific competition.

**Methods:**

We examined this trade-off in adult great knots *Calidris tenuirostris*, a molluscivorous long-distance migrating shorebird species, using interannual variation based on 2 years with different levels of food availability during their northward migratory staging in the northern Yellow Sea, China. We estimated individual home ranges and the extent of spatial overlap of home ranges of individually tagged birds in 2012 and 2015, whilst discounting for possible differences in body size, body mass, sex and migration schedule between years.

**Results:**

We found that home range size was not associated with body mass, arrival date, body size, or sex of the individual. Despite a significant difference in food availability between the two study years, there was no significant change in the 50% and 95% home range size of great knots in the contrasting situations. However, there was a significantly smaller spatial overlap between individuals in the year when food was less available, suggesting that great knots operated more independently when food was scarce than when it was abundant.

**Conclusions:**

These results suggest that minimizing intraspecific competition became more important when food was scarce. Where it is impossible to monitor all habitats *en route*, monitoring the local movements of shorebirds may offer a way to detect changes in habitat quality in real time.

**Supplementary Information:**

The online version contains supplementary material available at 10.1186/s40462-023-00427-9.

## Background

Spacing patterns of animals are thought to reflect trade-offs between the benefits of aggregation (for foraging, predator avoidance, mating, locomotion, and social learning; [1–9]) and the costs of aggregation (notably the competition for resources [10–13]). The more abundant the *per capita* food stock, the weaker the competition for food [10, 11]. Low costs of aggregating would enable conspecifics to live closely together and enjoy the benefits of aggregation [14]. How do animals respond in terms of aggregation when food availability is low?

One possibility is that conspecifics will use social foraging information to find hidden food, thus reducing search time and energy expenditure [1–4]. However, the resulting larger groups also increase resource competition, an increasing fraction of individuals will obtain too little food if the resources are limited [9, 12]. In such cases, animals may choose to forage in more dispersed ways, thus increasing their own chances of obtaining food [15]. The responses of animals’ aggregation (e.g., home range and distance from each other) to food scarcity remain unstudied.

Migratory shorebirds have faced severe food declines in some crucial staging areas in the Yellow Sea [16, 17]. The sudden and strong (> 95%) decline in shellfish availability in the Yalu Jiang Estuary National Nature Reserve (hereafter Yalu Jiang), Liaoning Province in northern China between the boreal springs of 2012 and 2013 resulted in dramatic decreases in intake rates and stark changes in the diet composition of shorebirds [18]. Nevertheless, at least initially, the abundance of staging shorebirds remained at similar levels, suggesting that birds had no alternative staging areas to go to [17]. A food supplementation experiment confirmed the shortage of food at Yalu Jiang [16].

The stark changes in food abundance at Yalu Jiang presented a contrasting ecological context [19] that enabled an assessment of how food abundance and other factors affect local space use and aggregation behavior in individuals of a group-living shorebird species [20]. For example, larger individuals may be more competitive, occupy better habitats, and have a smaller range of movement [21]. Female bar-tailed godwits (*Limosa lapponica*) prefer to move to areas with more worms, while males go to areas with relatively more molluscs to feed upon [22]. Earlier arrivals are more likely to occupy better habitats and thus not need to go on a wider search for food [23]. Thus, food competition may not be obvious when food is abundant but becomes apparent when food is scarce [10, 11]. We tested the prediction that home-range overlap will decrease when food availability is low.

In this study, we analyzed how a reduction in food availability would affect space use of radio-tagged great knots (*Calidris tenuirostris*), a molluscivorous, tactile-feeding, long-distance migrant that aggregates in large flocks during the non-breeding season, and mainly forages on molluscs such as the bivalves *Potamocorbula laevis* and *Mactra veneriformis,* and the gastropod *Umbonium thomasi* in Yalu Jiang [16, 17], we accounted for possibly associated, morphological and schedule variables (e.g. wing length, head + bill length, tarsus length, body mass and arrival date). A stark difference in food abundance occurred during two years of northward migration staging in Yalu Jiang [17, 24, 25]. To assess the degree of aggregation, we calculated the extent of overlap in the home range between tracked individuals. A high overlap would mean that individuals utilize similar places and suggest high levels of aggregation.

## Methods

### Study site

**Fig. 1:**
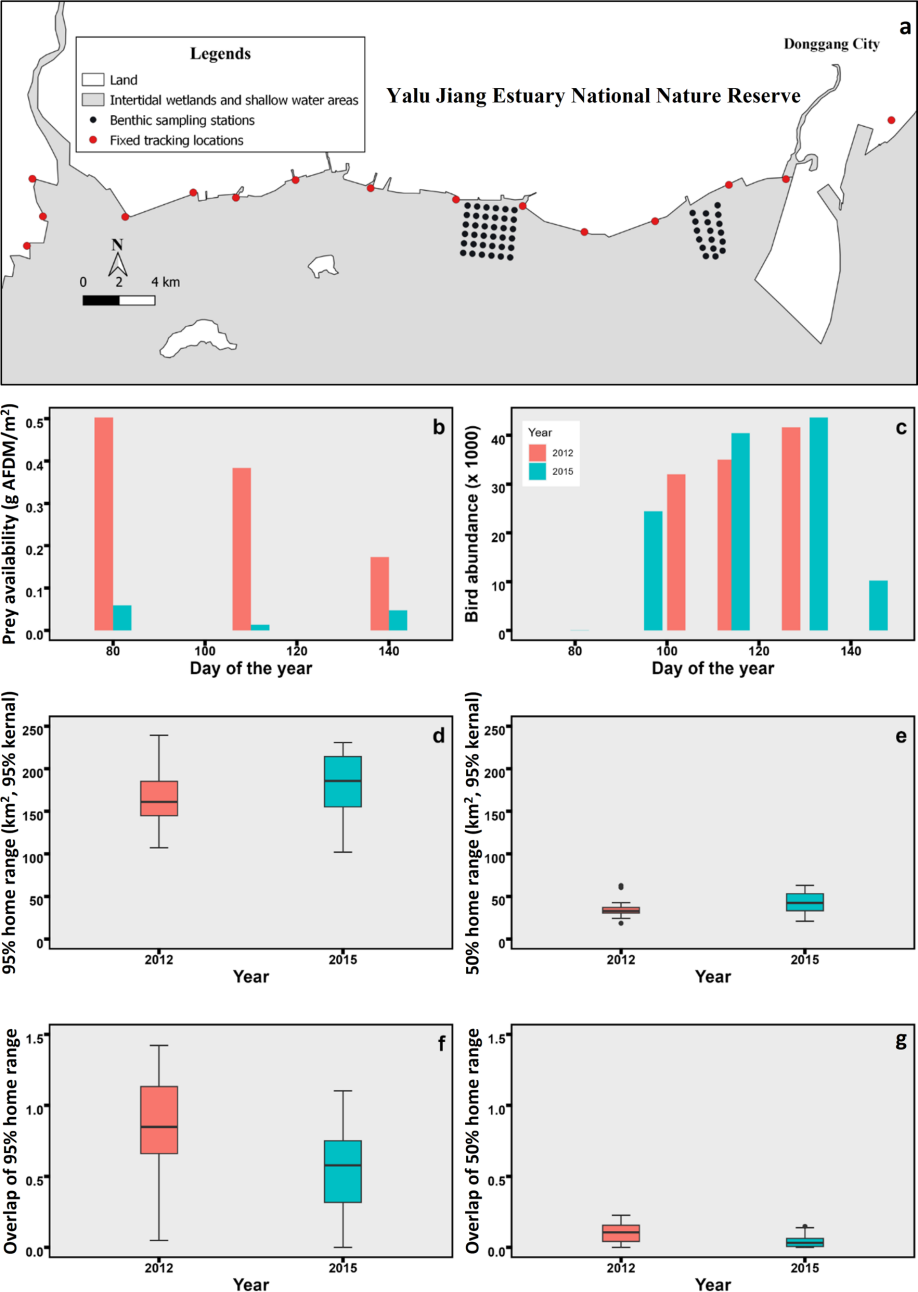
**a** Map of the Yalu Jiang National Nature Reserve. The variation in food availability (**b**), bird abundance (**c**), 95% home range (**d**), 50% home range (**e**), overlap of 95% home range (**f**), and overlap of 50% home range (**g**). Red boxes represent 2012, blue boxes represent 2015, and the asterisks are the outliers

During the non-breeding season, coastal shorebirds tend to live in flocks and are limited to foraging on intertidal flats [26]. Great knots eat mostly molluscs outside their breeding grounds, and their diet is relatively easy to quantify [5, 27, 28]. We radio-tracked the movements of great knots at Yalu Jiang (Fig. 1a), the northern Yellow Sea, China (39°40′–39°58′ N, 123°34′–124°07′ E). This site supported more than 50,000 great knots, and is one of the most important refuelling sites for the species during northward migration in the East Asian-Australasian Flyway [29, 30]. They forage on the intertidal mudflats, but roosts are variable. During neap high tides when the tidal mudflats are not completely submerged, great knots roost on the exposed mudflats. During spring high tides, when the tidal mudflats are completely submerged, great knots will roost in undeveloped land or on the banks of fish ponds due to the disappearance of the natural supratidal habitat as a result of land-claim [29]. As the final staging area before the flight to the breeding areas [30], Yalu Jiang is used by great knots for about two months (March–May), during which time they can double their body mass [31, 32].

### Bird abundances

Five counts were conducted at spring tides from mid-March to mid-May in both 2012 and 2015. Each count took place over the 2–3 days necessary to cover all 16 pre-roosts in the upper intertidal flats last covered by the incoming tide [17, 29]. The reason for not counting at the high-tide roosts is that these roosts in Yalu Jiang are scattered across extensive areas of fishponds, and availability may change frequently according to the water level in the ponds, making surveys less practical and accurate.

### Food availability

Benthic food items were sampled at grids of 36 stations with 500 m spacing at the main foraging area and at another 12 stations in the east foraging area of Yalu Jiang in both 2012 and 2015 (Fig. 1a; [17, 33]). Each sampling station was visited once per month from March to May. At each station, a sediment core with a 155 mm diameter covering 0.019 m^2^ was taken to a depth of 20 cm and washed over a 0.5 mm sieve. In the laboratory, fresh benthic organisms were identified and measured, and some of them were collected randomly to measure ash-free dry mass (hereafter AFDM; [18, 27]). We quantified the diet of great knots by observing foraging behavior and analyzing droppings based on local studies in 2011, 2012, 2016 and 2017 [18, 27]. AFDM was estimated from length measurements for each species to evaluate the biomass of all the potential prey [18, 27]. Total food availability was the summed biomass of all recorded prey species each month or year (as presented earlier in [18, 27]).

### Radio tracking

Chongming Dongtan, Shanghai, located 1039 km south of Yalu Jiang (39°40′–39°58′ N, 123°34′–124°07′ E), is an important stopover site for migratory waterbirds, including great knots. During northward migration, great knots often make a brief stop at Chongming Dongtan before moving to key staging sites further north in the Yellow Sea, including Yalu Jiang [25, 30]. Great knots were captured using clap nets on the intertidal flats at both Chongming Dongtan and Yalu Jiang in 2012 as part of regular shorebird banding. At Chongming Dongtan, over 500 great knots were banded in 2012, of which 40 adults in six groups, at 5–6 days intervals, were randomly selected to be fitted with radio transmitters. Of these 40 birds, 12 were subsequently detected at Yalu Jiang within the same season [32]. At Yalu Jiang, 10 additional adults were randomly selected from three groups captured at intervals of 10 days and tagged (Table 1), [24, 25, 32]. Of these birds, 22 great knots provided tracking data at Yalu Jiang in 2012. In 2015, over 500 great knots were caught at Chongming Dongtan, of which 72 adult individuals were selected randomly and tagged. Subsequently, 22 stopped at Yalu Jiang. In 2015, no great knots were tagged at Yalu Jiang (Table 1).

**Table 1:** The banding locations and numbers of radio-tagged great knots in 2012 and 2015

Number of great knots	2012	2015
Banded at Chongming Dongtan	> 500	> 500
Radio-tagged at Chongming Dongtan	40	72
Radio-tagged and subsequently recorded at Yalu Jiang	12	22
Banded at Yalu Jiang	> 300	0
Radio-tagged at Yalu Jiang	10	0
Total number of radio-tagged birds recorded at Yalu Jiang	22	22
Total number of triangulated fixes recorded	1522	1186
Total number of triangulated fixes used for analysis	1427	1023

**Table 2:** The candidate models to explain the 95% home range and 50% home range of staging great knots in Yalu Jiang. ‘Sex’ was only included in 2012 data.

Models	*df*	AIC_C_	△AIC
*95% home range 2012*			
95% home range ~ Tarsus length + Body mass + Wing length	4	384.3	–
95% home range ~ Sex + Body mass + Wing length	4	386.0	1.68
95% home range ~ Body mass + Wing length	2	386.2	1.90
95% home range ~ Body mass	1	386.2	1.94
95% home range ~ Sex + Tarsus length + Body mass + Wing length	5	386.3	1.98
*50% home range 2012*			
50% home range ~ Body mass + Wing length	3	338.2	–
50% home range ~ Body mass	2	339.2	0.91
50% home range ~ Head + bill length + Body mass + Wing length	4	339.2	0.95
50% home range ~ Arrival date + Body mass	3	339.7	1.48
50% home range ~ Arrival date + Body mass + Wing length	4	340.3	2.03
*95% home range 2012 + 2015*			
95% home range ~ Body mass	2	658.7	–
95% home range ~ Arrival date + Body mass	3	659.6	0.85
95% home range ~ Body mass + Year	2	659.6	0.89
95% home range ~ Arrival date + Body mass + Year	4	660.8	2.04
95% home range ~ Tarsus length + Body mass	3	660.9	2.16
*50% home range 2012 + 2015*			
50% home range ~ Arrival date + Body mass + Year	4	582.9	–
50% home range ~ Body mass + Year	3	583.6	0.73
50% home range ~ Arrival date + Body mass	3	583.8	0.93
50% home range ~ Body mass	2	584.8	1.96
50% home range ~ Wing length + Body mass + Year + Arrival date	5	585.2	2.35

Top 5 models of each analysis were showed

AIC_C_: corrected AIC values for small sample sizes; △AIC: the difference in AIC_C_ between each model and the best model with the lowest AIC_C_

In all cases, captured individuals were measured, weighed, and banded, and the body mass (measured to the nearest 0.1 g), wing length (0.1 cm), head + bill length (0.1 cm), and tarsus length (0.1 cm) were recorded. In 2012, we pulled 2–3 body feathers from each tagged bird from which DNA was extracted, and molecular techniques were used to sex the birds [32]. Very High Frequency (VHF) transmitters (Holohil Systems, 2.45 g, < 2.3% of body mass, battery life > three months) were applied by gluing them (Locktite 454; Henkel) to an area of clipped feathers on the lower back of the birds [32].

During tracking, we set 16 fixed tracking stations along the seawall every 5 km (Fig. 1a), and several tracking stations were strategically placed along the coast. Two people 500 m apart scanned for signals using a Yagi 3-element folding antenna, a TRX-2000S radio receiver (Wildlife Materials International, Inc.) and a compass (bearing measured to 1°). We used triangulation to locate individual great knots [24]. From mid-March to late May 2012, we tracked the tagged birds from 0700 to 1800 h at Yalu Jiang, except on rainy days (4 rainy days out of 77 tracking days). All the fixed tracking stations were visited (approximately 30–45 min per station) twice every day, once during the rising tide and once during the falling tide. Bird activity is affected by the tide, so to avoid sampling bias, the tracking route of observers was different every day, but it was designed so that each fixed tracking station was visited during high, mid, and low tide within each week. After the tide covered the mudflats and the birds were forced to fly to roost on the banks of aquaculture ponds [29], it was difficult for fixed stations to cover all the ponds due to the barrier of the aquaculture banks, so we selected some stations at random (Distance between neighboring stations is less than 5 km) for tracking. Location fixes were omitted from the analyses if the direction detected was unclear due to a weak (low sound and low dashboard oscillation from the receiver) signal (e.g., long distance, moving bird). In addition, consecutive fixes within 30-min intervals for the same bird were excluded to avoid bias from short-term sampling on the estimation of the home range (Sanzenbacher and Haig 2002). In total, 1522 location fixes were recorded in 2012, and 1427 (94%) were used for analysis, while 1186 were detected in 2015, and 1023 (86%) were used for analysis (Additional file 1: Table S1).

We used home ranges (95% kernel density estimate—the activity areas of birds, and 50% kernel density estimate—the core territory of birds) per individual to represent the space use of great knots using the package ‘*adehabitatHR*’ [34] in R [35]. Individuals with at least 30 fixes were used to estimate home ranges (n = 34, Table S1). The ‘href’ method (termed the ‘ad hoc’ method, smoothing parameter specified as h = 1000) was used to estimate the smoothing parameter for kernel estimation since it provides conservative density estimates that are especially useful when the number of observations is low [36, 37].

### Statistical analysis

To assess the factors affecting the home ranges (both 95% and 50%) of birds at Yalu Jiang, we used linear regressions to explore how home ranges were related to sex, body mass, wing length, head + bill length, tarsus length, arrival date and year of tagged great knots. Because sex was only determined in 2012, we first regressed home ranges against sex, body mass, wing length, head + bill length, tarsus length and arrival date to assess whether sex affected the home ranges of great knots. Then we conducted linear regression between home ranges and body mass, wing length, head + bill length, tarsus length, arrival date and year to assess what factors affected the home ranges in Yalu Jiang in both 2012 and 2015. The second-order Akaike’s Information Criterion (AICc) was used for evaluating the relative support of a prior candidate model [38] calculated with the package ‘*MuMIn’* [39] in R. Model-averaged estimates were computed based on all candidate models to assess the support of variables [40]. The function *model.avg* in package *MuMIn* [39] was used to assess the coefficients of the variables in models. If the 95% Confidence Interval (CI) of coefficient does not contain 0, then the effect of that variable is significant.

We used the overlap of 95% home range or 50% home range between individuals to represent the degree of aggregation of great knots. We estimated overlap in the home range of birds from neighboring individuals using the utilization distribution overlap index (UDOI; [41]). UDOI = 0 means that the space used between individuals does not overlap. UDOI = 1 means 100% overlap in the space used. When the two utilization distributions had a high degree of overlap in size outline but were not uniformly distributed in the main activity hotspots, UDOI values can be > 1 [41]. Calculations of percentage overlap and UDOI were performed by using the package ‘*adehabitatHR’* [34] in R.

Furthermore, since the number of times and timing of bird surveys and macrobenthic surveys were the same in the two years, paired Student’s t-test was used to compare the differences in bird abundance and food availability between 2012 and 2015. When comparing bird abundances, the five surveys for each of the two years were paired according to date. When comparing prey availability, the prey availability of different sampling stations in different months was paired year to year. Independent t-tests were used to compare the differences in the extent of the overlap of the 95% home range and the overlap of the 50% home range between 2012 and 2015. All analyses were run in R Studio version 4.1.2 in Windows [42].

## Results

In 2012, the average density of all available prey types was 0.35 ± 0.92 g AFDM/m^2^ (n = 144). In 2015, after the main food *P. laevis* had declined more than 90%, the average availability of all known prey types was significantly lower than that in 2012 (89% lower), with only 0.04 ± 0.09 g AFDM/m^2^ (n = 144) recorded (Fig. 1b, paired t-test, t = 4.05, df = 143, *p* < 0.001). The numbers of great knots counted in 2012 and 2015 were similar (paired t-test, t = − 0.69, df = 4, *p* = 0.53, Fig. 1c), with peak numbers of more than 40,000 birds in both years (Fig. 1c).

In 2012, the body mass of tagged great knots (with enough fixes to analyze home ranges, n = 20) was 147.6 ± 23.3 g, with a wing length of 190.5 ± 4.8 cm, head + bill length 74.9 ± 1.8 cm, tarsus length 36.4 ± 1.0 cm, and an arrival date of 12 April ± 9 days; the average 95% home range size of great knots was 167 ± 35 km^2^ and the 50% home range was 35 ± 11 km^2^. In 2015, the body mass of tagged great knots (with enough fixes to analyze home ranges, n = 14) was 136.6 ± 9.5 g, with a wing length of 193.9 ± 4.7 cm, head + bill length 75.5 ± 1.5 cm, tarsus length 36.6 ± 1.0 cm, and an arrival date of 11 April ± 7 days.

The overall averages of the two values of the home range were 182 ± 40 km^2^ (95%) and 43 ± 14 km^2^ (50%). Sex did not have any significant effect on both 95% and 50% (the slope β of the relationship of values are shown in Table 3) home range sizes in 2012 (Table 2). In the models assessing the home ranges in both years together, the home range sizes of great knots were not significantly affected by body mass, arrival date, wing length, head + bill length, tarsus length and year (all the slopes β are shown in Table 3).

**Table 3:** The results of model average of 95% Confidence Interval (CI) of coefficient (Slope β) for the variables in all AIC candidate models (see Table 2). The 95% Confidence Interval (CI) does not contains zero, the effect of that variable is significant

Home range	Variables	Slope β	95% CI	Significant
*2012 95% home range size*				
	Intercept	41,440.0	− 13,160.0–96,040.0	No
	Body mass	0.0	− 17.5–17.4	No
	Sex	− 682.8	− 2550.8–1185.2	No
	Arrival date	− 17.1	− 71.8–37.5	No
	Wing length	− 284.5	− 547.7–− 21.3	Yes
	Head + bill length	40.3	− 210.0–290.5	No
	Tarsus length	785.5	− 185.6–1756.6	No
*2012 95% home range size*				
	Intercept	12,766.8	− 1196.3–26,729.9	No
	Body mass	1.1	− 5.3–7.6	No
	Sex	3.5	− 389.5–396.6	No
	Arrival date	− 10.9	− 33.4–11.7	No
	Wing length	− 65.7	− 137.1–5.6	No
	Head + bill length	46.8	− 63.7–157.2	No
	Tarsus length	19.3	− 109.7–148.3	No
*2012 and 2015 95% home range size*				
	Intercept	− 347,200	− 1,080,600.0–386,200.0	No
	Body mass	− 7.6	− 30.5–15.2	No
	Arrival date	− 34.19	− 101.4–33.0	No
	Wing length	− 8.6	− 78.8–61.5	No
	Head + bill length	− 29.6	− 238.5–179.3	No
	Tarsus length	99.8	− 295.1–494.7	No
	Year	183.4	− 182.4–549.2	No
*2012 and 2015 95% home range size*				
	Intercept	− 315,500	− 661,200.0–30,200.0	No
	Body mass	− 2.1	− 9.3–5.1	No
	Arrival date	− 27.0	− 57.6–3.7	No
	Wing length	− 4.0	− 28.8–20.8	No
	Head + bill length	− 11.9	− 81.5–57.7	No
	Tarsus length	− 3.4	− 109.5–102.7	No
	Year	161.0	− 11.4–333.4	No

**Fig. 2:**
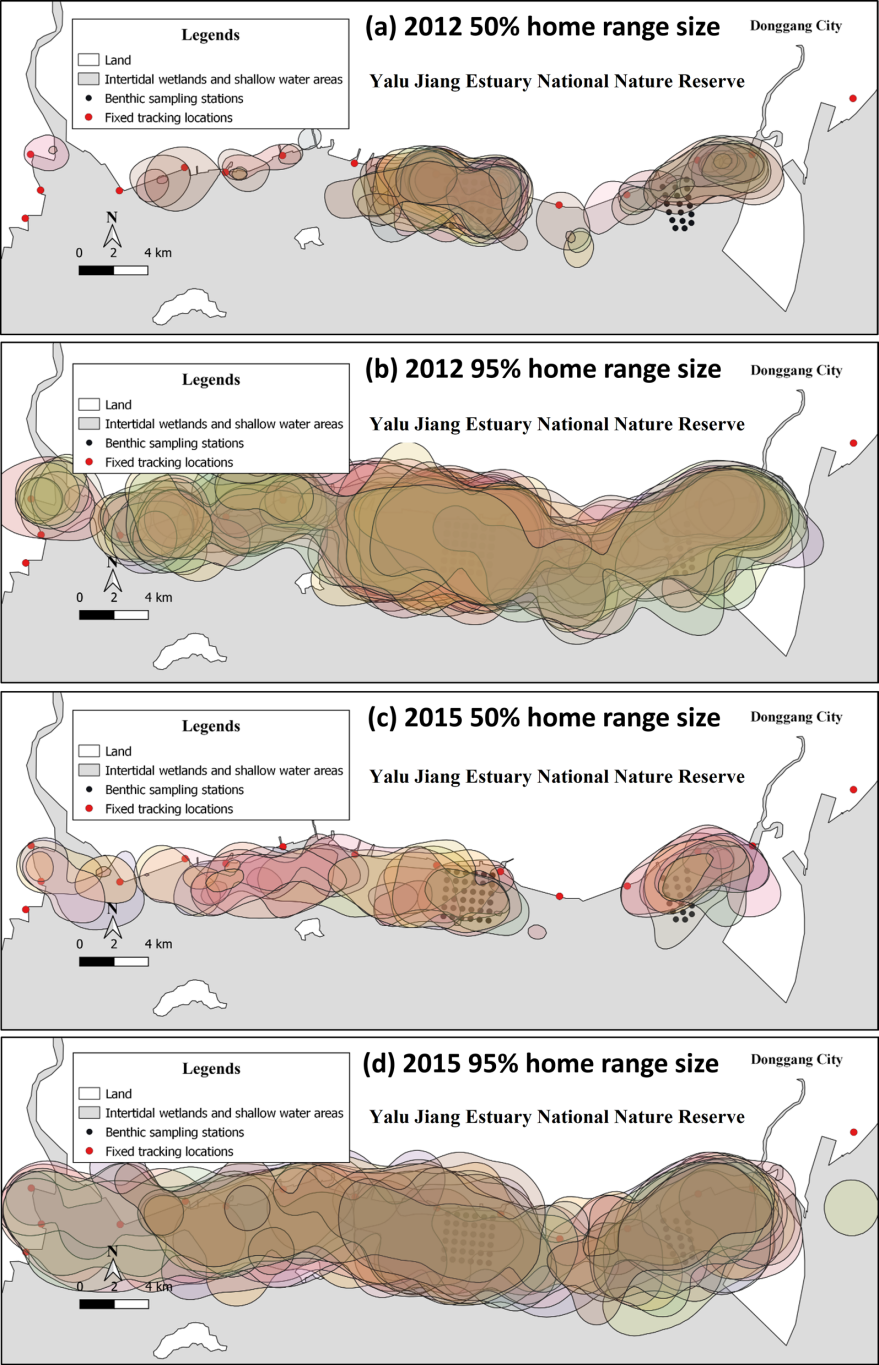
Fifty percent (**a**, **c**) and 95% (**b**, **d**) home range sizes of tracked great knots in 2012 (**a**, **b**) and 2015 (**c**, **d**) at Yalu Jiang. Home ranges with different colors indicate the home range size of different individuals

Despite this similarity of home range size in a food-rich (2012) and a food-poor (2015) year, the overlap (UDOI) of the 95% home range was significantly larger in 2012 (0.87 ± 0.30, n = 190) than in 2015 (0.55 ± 0.28, n = 91) (t = 11.9, df = 560, *p* < 0.001). Equally, the overlap of the 50% home range was also significantly larger in 2012 (0.10 ± 0.06, n = 190) than in 2015 (0.04 ± 0.04, n = 91) (Fig. 1f, g, Fig. 2; t = 12.4, df = 560, *p* < 0.01).

## Discussion

This study showed that the home range size of great knots was not associated with body mass, arrival date, sex, wing length, head + bill length, or tarsus length of birds. Although the food available to great knots in 2015 was only 11% of that in 2012 in Yalujiang, there is no significant difference between the average 50% and 95% home range size between years. However, the spatial segregation of individuals, expressed as the overlap between individual home ranges, was significantly lower in the low-food year 2015 than in 2012. This is consistent with the prediction that levels of aggregation, thus the extent of overlap in home ranges, would decrease as food becomes scarce [12].

In 2015, the year with low food abundance, the 50% and 95% home range of the great knots was not significantly different from that in 2012, indicating that the available habitats in Yalu Jiang might be fully utilized by shorebirds with little space for expansion. Nevertheless, this does not mean that great knots did not try to expand their home ranges: both 50% and 95% home range sizes were slightly larger in 2015 than in 2012, hinting that individual great knots did try to visit more intertidal area in limited total range where they commuted and searched for food. This is also consistent with the distribution of bird counts, where in 2012 birds were recorded at a handful of sites, and by 2015 the birds became more dispersed (Additional file 1: Figure S1). After the severe food decline, the core foraging area of great knots in Yalu Jiang was still located in the middle segment of the reserve (Fig. 1a; [18]), but more individuals foraged in adjacent areas [16].

In 2012, when food availability was high (Fig. 1B), competition might have been low enough that large aggregations would not lead to high intraspecific competition [27] and instead, may have facilitated food finding [1, 2]. With the 89% decline in mollusc food in 2015, great knots showed significantly smaller overlap with conspecifics. A food supplementation study in 2018 (a year of food shortage such as 2015) showed that supplemental food rapidly attracted nearly half of the total number of great knots at Yalu Jiang. They consumed over 90% of the supplemental food in a short time [16], suggesting that competition for food did become intense at Yalu Jiang. To what extent avoidance of intraspecific competition overrode any gains of stronger aggregative behavior needs to be studied further [43–45].

When food became scarce in Yalu Jiang, great knots did not increase their home range size, possibly due to a lack of suitable habitat. Instead, staging great knots reduced the overlap of home ranges as food availability decreased. Such a change in movement pattern could reduce intraspecific competition [12] and allow the search for more sparsely distributed food. This change from foraging in large groups to foraging in smaller dispersed groups, and associated changes in social behavior and local movement patterns of great knots could indicate a reduction in food abundance. Understanding how migratory shorebirds respond behaviourally to changes in habitat quality, allows for the timely identification of potential changes in habitat quality through monitoring of bird movements and the timely and effective development of conservation measures when it is not possible to fully monitor the quality of all habitats *en route* along the entire flyway.

### Supplementary Information

**Additional file 1.** **Table S1.** Number of location fixes detected at Yalu Jiang for radio-tracked great knots in 2012 and 2015; only fixes > 30 for a bird are shown. **Figure S1.** The distribution of great knots recorded in the bird count in 2012 and 2015, only the highest number was shown for each month.

## Data Availability

The original data used in the study are included in the electronic supplementary material.
