# Supplementary material for: Individuals of a group-living shorebird show smaller home range overlap when food availability is low
Source: Mov Ecol. 2023 Oct 27;11:70. doi: 10.1186/s40462-023-00427-9 (PMC10612227; doi:10.1186/s40462-023-00427-9)
Supplement: Supplementary file 1 — Additional file 1. Table S1. Number of location fixes detected at Yalu Jiang for radio-tracked great knots in 2012 and 2015; only fixes > 30 for a bird are shown. Figure S1. The distribution of great knots recorded in the bird count in 2012 and 2015, only the highest number was shown for each month. [file 40462_2023_427_MOESM1_ESM.docx]

Individuals of a group-living shorebird show smaller home range overlap when food availability is low

He-Bo Peng^1,2^, Chi-Yeung Choi^3,4,5*^, Zhijun Ma^6^, Allert I. Bijleveld^2^, David S. Melville^7^, Theunis Piersma^2,3,8,9^

Table S1. Number of location fixes detected at Yalu Jiang for radio-tracked great knots in 2012 and 2015; only fixes > 30 for a bird are shown.

| Year | Bird (tag reference) | Number of fixes |
| --- | --- | --- |
| 2012 | B12-01 | 108 |
|  | B12-03 | 83 |
|  | B12-04 | 68 |
|  | B12-05 | 78 |
|  | B12-07 | 72 |
|  | B12-08 | 42 |
|  | B12-09 | 93 |
|  | B12-10 | 54 |
|  | B12-11 | 72 |
|  | B12-12 | 75 |
|  | B12-13 | 48 |
|  | B12-15 | 118 |
|  | B12-16 | 69 |
|  | B12-17 | 71 |
|  | B12-18 | 67 |
|  | B12-19 | 76 |
|  | B12-20 | 57 |
|  | B12-21 | 69 |
|  | B12-22 | 60 |
|  | B12-23 | 47 |
| 2015 | B15-140s | 94 |
|  | B15-180s | 55 |
|  | B15-270s | 93 |
|  | B15-351s | 67 |
|  | B15-421s | 93 |
|  | B15-461s | 76 |
|  | B15-480s | 70 |
|  | B15-510s | 52 |
|  | B15-591s | 87 |
|  | B15-691s | 78 |
|  | B15-711q | 43 |
|  | B15-711s | 37 |
|  | B15-751s | 99 |
|  | B15-820s | 78 |


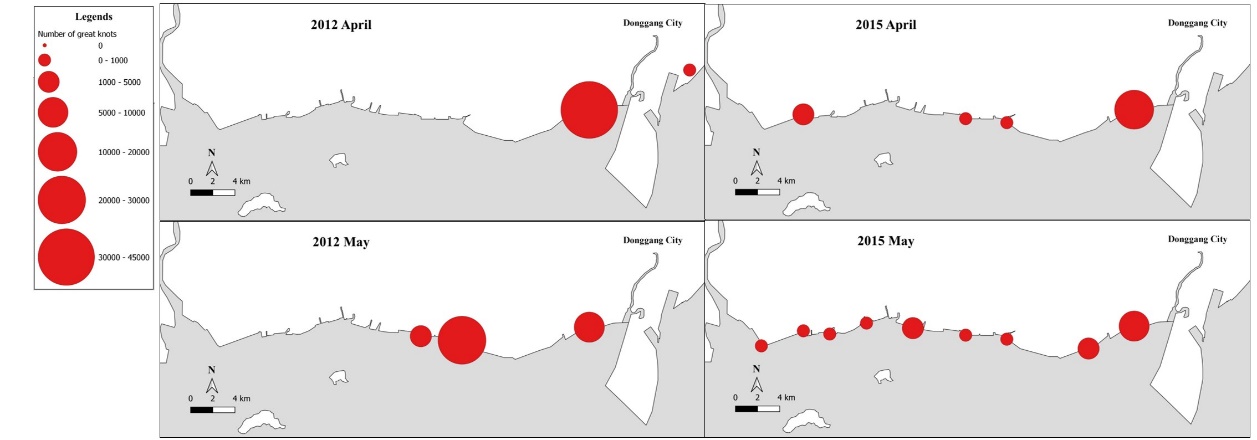


Figure S1. The distribution of great knots recorded in the bird count in 2012 and 2015, only the highest number was shown for each month.
